# Improving Access to Menopause Healthcare for Women with Intellectual Disabilities: A Quality Improvement Project in Oxleas NHS Foundation Trust

**DOI:** 10.1192/bjo.2026.11456

**Published:** 2026-06-30

**Authors:** Alisha Patel

**Affiliations:** Oxleas NHS Foundation Trust, London, United Kingdom

## Abstract

**Aims:**

To improve access to menopause healthcare for women with intellectual disabilities (ID) by August 2026 in Oxleas NHS Foundation Trust, by providing education and person-centred services. Objectives were for 90% of clinicians to feel confident in identifying menopause symptoms; perimenopause screening tool implementation; and development of menopause groups for patients and carers with attendees improving by at least one point using Likert scales.

**Methods:**

Gynaecologist-led teaching on perimenopause and menopause in women with ID was delivered to Greenwich Learning Disability Team.

A baseline audit established perimenopause screening of women aged over 40 at psychiatric assessments in Greenwich Learning Disability Team. The Greene Climacteric Scale was developed into an easy-read perimenopause screening tool following Plan–Do–Study–Act (PDSA) methodology with patient and clinician collaboration. This tool and a prompt on the Psychiatry assessment template were implemented.

Menopause Groups were developed for women with ID, carers and relatives, using PDSA cycles with patient feedback. Groups addressed physical and emotional symptoms of perimenopause and menopause, treatment options and coping strategies, over three 1.5-hour weekly workshops. Practical activities included menstrual pads, flash cards and mindfulness alongside sharing of experiences.

**Results:**

31 clinicians attended menopause teaching and 100% deemed menopause as relevant to clinical practice with knowledge of perimenopause/menopause in PwID increasing from 9% to 70%. Confidence in discussing symptoms with patients increased from 57% to 80% and awareness of services increased from 17% to 90%. Thematic analysis revealed barriers included embarrassment, lack of knowledge, diagnostic overshadowing and poor patient understanding.

Of the 7 eligible patients from the baseline audit, none were screened for perimenopause, menopause or menstruation status between August 2024–August 2025.

6 patients and 6 carers attended the Greenwich Menopause Group. On a 3-point Likert Scale, all patients improved by >1 point. Knowledge of symptoms increased from 50% to 100% and confidence in getting help increased from 67% to 100%. On a 5-point Likert Scale, carers’knowledge increased from 4.3 to 4.8. Confidence in talking to their patients increased from 4.5 to 4.8. Patients enjoyed visual demonstrations and wellbeing activities, and carers appreciated open discussions and easy-read materials. Multi-disciplinary facilitators felt it was a rewarding and valuable initiative.

**Conclusion:**

Menopause groups, screening and upskilling staff to support women with ID to navigate the menopause has improved the quality of patient care. Collaboration and dissemination of interventions across South East London and North London ID services, Primary Care and Mencap is ongoing to widen access.

